# A chromosomal analysis of eleven species of Gyrinidae (Coleoptera)

**DOI:** 10.3897/CompCytogen.v10i1.7662

**Published:** 2016-03-21

**Authors:** Robert B. Angus, Teresa C. Holloway

**Affiliations:** 1 Division of Life Sciences (Insects), The Natural History Museum, Cromwell Road, London SW7 5BD, UK; 2 School of Biological Sciences, Royal Holloway University of London, Egham Hill, Egham, Surrey TW20 0EX, UK

**Keywords:** Coleoptera, Gyrinidae, *Gyrinus*, *Orectochilus*, chromosomes, karyotypes, C-banding

## Abstract

Karyotypes are presented for 10 species of *Gyrinus* Geoffroy, 1762: *Gyrinus minutus* Fabricius, 1798, *Gyrinus caspius* Ménétriés, 1832, *Gyrinus paykulli* Ochs, 1927, Gyrinus distinctus Aubé, 1836 var. fairmairei Régimbart, 1883, *Gyrinus marinus* Gyllenhal, 1808, *Gyrinus natator* (Linnaeus, 1758), *Gyrinus opacus* Sahlberg, 1819, *Gyrinus substriatus* Stephens, 1869, *Gyrinus suffriani* Scriba, 1855, *Gyrinus urinator* Illiger, 1807 and for *Orectochilus villosus* (Müller, 1776) (Coleoptera: Gyrinidae). The 10 *Gyrinus* species have karyotypes comprising 13 pairs of autosomes plus sex chromosomes which are X0 (♂), XX (♀), with the X chromosomes the longest in the nucleus. *Orectochilus villosus* has 16 pairs of autosomes plus X0, XX sex chromosomes. The data obtained by Saxod and Tetart (1967) and Tetart and Saxod (1968) for five of the *Gyrinus* species are compared with our results. Saxod and Tetart considered the X chromosome to be the smallest in the nucleus in all cases, and this is considered to result from confusion arising from uneven condensation of some of the chromosomes. Small differences between the chromosomes of different *Gyrinus* species have been detected, but not between Greenland and Swedish populations of *Gyrinus opacus*, nor between typical *Gyrinus distinctus* from France and Gyrinus distinctus var. fairmairei from Kuwait.

## Introduction

The Gyrinidae appear to be the first coleopteran family to be subjected to chromosomal analysis using air-drying of inflated cells on glass slides (Saxod and Tetart 1967, Tetart and Saxod 1968). The first account of an acetic acid dissociation, air-drying technique for use on insect cells was by Crozier (1968). He used hypotonic saline to inflate living cells prior to fixation, whereas Tetart (1969) began by dissecting out gonads in an isotonic saline solution and placing small pieces of tissue on slides and there fixing them briefly, with a fixative comprising absolute alcohol (70%) and glacial acetic acid (30%). After a minute or so the fixative is poured off and replaced with a swelling solution comprising 40% absolute alcohol, 30% glacial acetic acid and 30% distilled water. This causes tissue swelling and cell dissociation, monitored under a microscope. Additional swelling solution may be added, and mechanical dissociation of the tissue may be needed. The slide is then dried over a spirit lamp and fresh fixative is added. Treatment with hydrochloric acid (concentration and time not given) prevents cytoplasmic staining, and the chromosomes are then stained with Giemsa.

This technique is clearly different from Crozier’s hypotonic inflation of living cells, which is the basis for all subsequent air-drying techniques used on insects, but has produced some very good clear chromosome spreads for use in karyotype preparation, not least in five species of *Gyrinus* Geoffroy, 1762.

This background information provided a framework to assess the chromosomes obtained from a sample of living *Gyrinus opacus* C.R. Sahlberg, 1819, from Greenland, sent by B.O. Svensson in 1995. The first obvious result of the analysis was the discovery that *Gyrinus opacus* has an X chromosome much larger than was reported by Saxod and Tetart in any of the five species they studied. Further work by Teresa Holloway in 2006, as a final-year undergraduate project at Royal Holloway, University of London, forms the basis of this paper and shows that Saxod and Tetart were in fact mistaken in their belief that the X chromosome of *Gyrinus* species was the smallest in the nucleus, though they were correct in stating that the karyotypes comprised 13 pairs of autosomes and an X0 sex chromosome system.

## Material and methods

**Figures 1–4.**
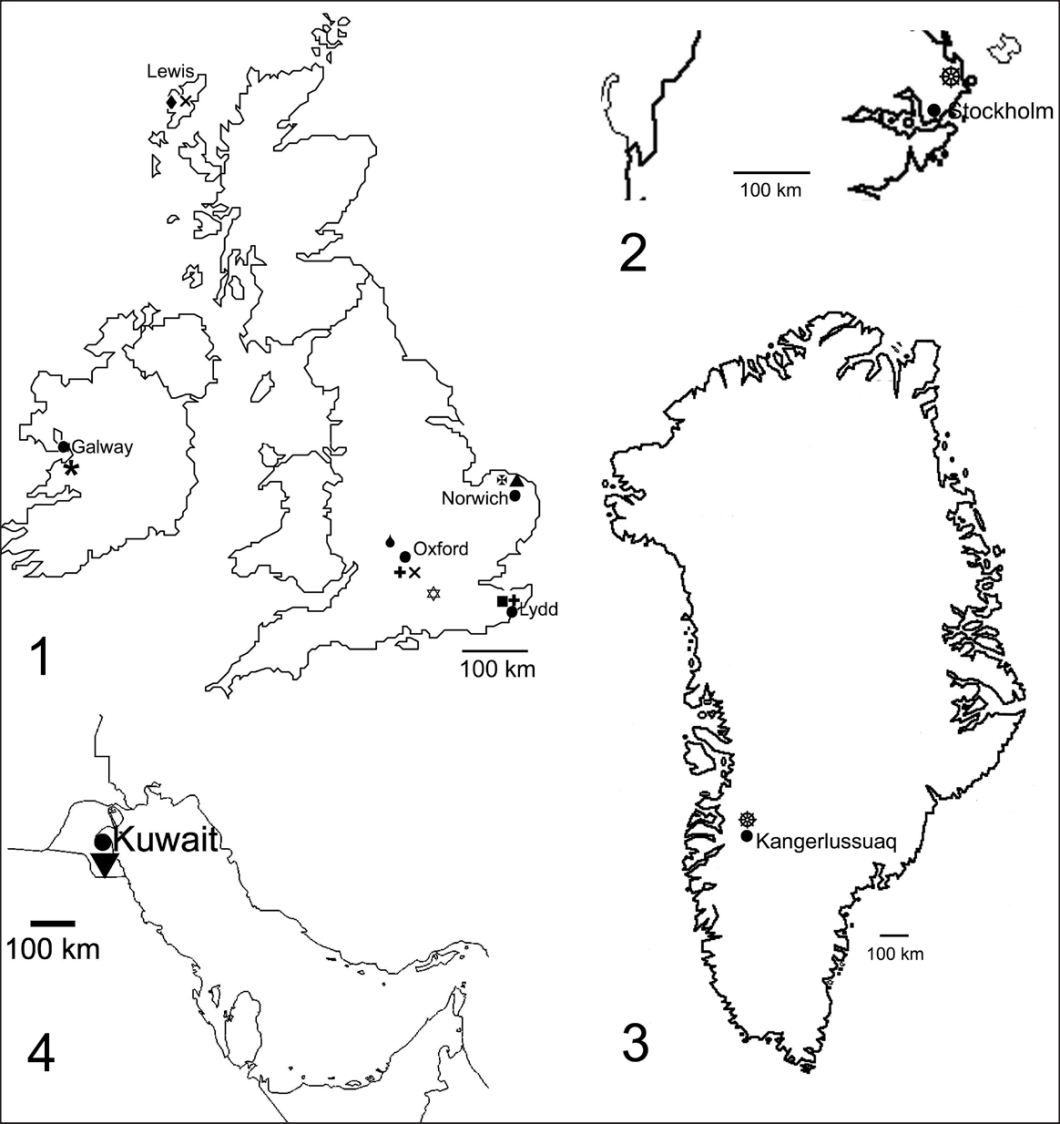
Maps showing the localities of the material studied. **1** British Isles for *Gyrinus minutus*, *Gyrinus caspius*, *Gyrinus paykulli*, *Gyrinus marinus*, *Gyrinus natator*, *Gyrinus substriatus*, *Gyrinus suffriani*, *Gyrinus urinator* and *Orectochilus villosus* **2** Stockholm area of Sweden for *Gyrinus opacus* **3** Greenland for *Gyrinus opacus* **4** Kuwait for *Gyrinus distinctus fairmairei*. For symbols see Table 1.

Details of the material analysed, including the geographical source, number and sex of the specimens are given in Table 1. The localities from which the material was obtained are shown in Figs 1–4. Living adults from Greenland, Sweden and Kuwait were kept in covered aquaria and were given living adult fruit-flies (*Drosophila*), thrown down on to the surface film of the water, as food. Chromosome preparations were made from mid-gut, testis and ovaries, using the methods described by Dutton and Angus (2007). The methods of C-banding and photography, and assemblage of karyotypes were also as given by Dutton and Angus. Relative Chromosome Lengths (RCL, the length of each chromosome expressed as a percentage of the total haploid autosome length (i.e. not counting the sex chromosomes) in the nucleus) are given as approximate values, without any statistical analysis–the data are insufficient for statistical testing.

**Table 1.** Material used for chromosome analysis.

Species	Locality	Map	Specimens analysed
*Gyrinus minutus* Fabricius, 1798	SCOTLAND: Isle of Lewis	Fig. 1, ♦	1 ♂
*Gyrinus caspius* Ménétriés, 1832	ENGLAND: Kent, Lydd	Fig. 1, ■	1 ♂
*Gyrinus paykulli* Ochs, 1927	ENGLAND: Norfolk, Catfield Fen	Fig. 1, ✠	1 ♂
Gyrinus distinctus Aubé, 1836 var. fairmairei Régimbart, 1883	KUWAIT: Ras Az Zawr district.	Fig. 4, ▼	1 ♂
*Gyrinus marinus* Gyllenhal, 1808	ENGLAND: Kent, Lydd;	Fig. 1, **+**	1 ♂
	Oxfordshire, Kennington	Fig. 1, **+**	1 ♂
*Gyrinus natator* (Linnaeus, 1758)	IRELAND: Galway, Lough Briskeen	Fig. 1, ★	1 ♂
*Gyrinus opacus* Sahlberg, 1819	SWEDEN: Upland, Vädö	Fig. 2, ⎈	2♂♂, 1♀
	GREENLAND: Kangerlussuaq	Fig. 3, ⎈	2 ♂♂, 1♀
*Gyrinus substriatus* Stephens, 1869	ENGLAND: Oxfordshire, Kennington	Fig. 1, ✖	2 ♂♂
	SCOTLAND: Isle of Lewis	Fig. 1, ✖	1 ♂
*Gyrinus suffriani* Scriba, 1855	ENGLAND: Norfolk, Catfield Fen	Fig. 1, ▲	1 ♂
*Gyrinus urinator* Illiger, 1807	ENGLAND: Surrey, Tilford	Fig. 1, ✡	4 ♂♂, 2 ♀♀
*Orectochilus villosus* (Müller, 1776)	ENGLAND: Oxfordshire, Stonesfield, River Evenlode	Fig. 1, 🌢	1 ♂, 1 ♀

## Results

### *Gyrinus*

The karyotypes of all 10 species included here are broadly similar, with 2n = 26 + X0 (♂), and 26 + XX (♀). The autosomes are mainly either metacentric or submetacentric, and their RCLs range from about 11 to about 6. The X chromosome is metacentric and the largest in the nucleus, with RCL normally ranging from about 12–16. C-banding, where known, is confined to the centromere regions.

#### Subgenus *Gyrinulus* Zaitzev, 1907

##### *Gyrinus minutus* Fabricius, 1798

**Figure 5.**
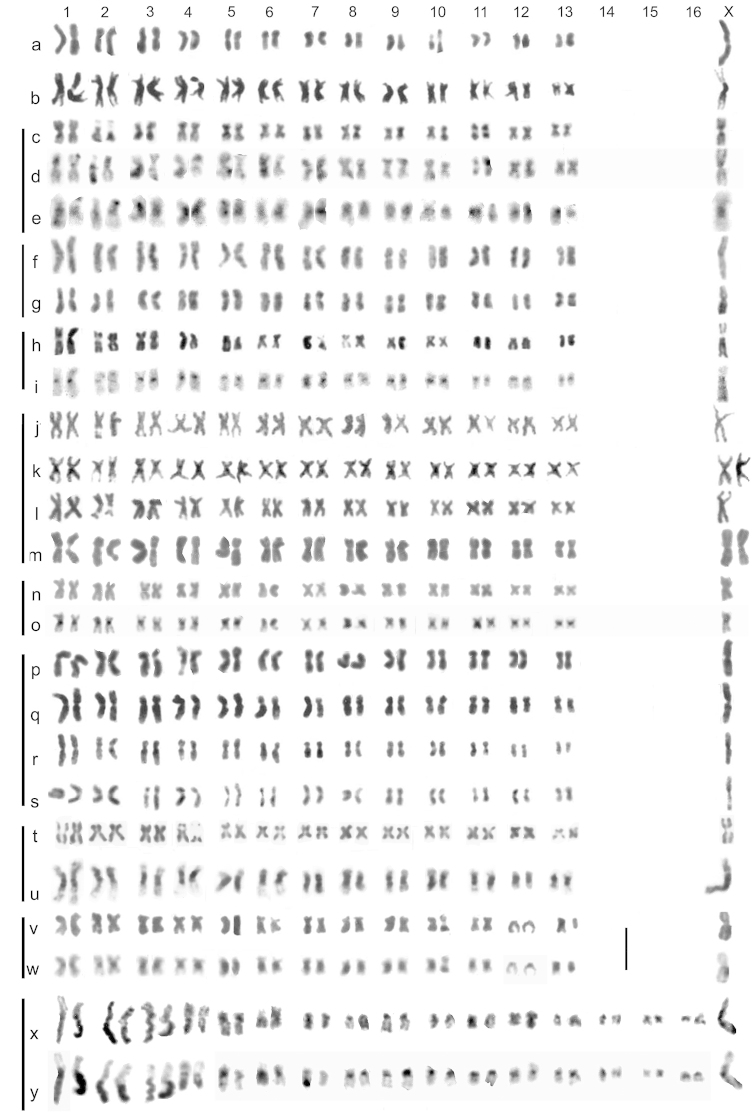
Mitotic chromosomes of Gyrinidae, arranged as karyotypes. **a** *Gyrinus minutus*, ♂, Isle of Lewis, testis, plain (Giemsa stained) **b** *Gyrinus caspius*, ♂, Lydd, mid-gut, plain **c–e** *Gyrinus paykulli*, ♂, Catfield Fen, mid-gut **c** plain **d** partially C-banded, still showing chromosome morphology, **e** the same nucleus fully C-banded, much chromosome morphology lost **f, g** *Gyrinus distinctus fairmairei*, ♂, Kuwait, testis, plain **h, i** *Gyrinus marinus*, ♂, Lydd, testis **h** plain **i** the same nucleus C-banded **j**, **k** *Gyrinus opacus*, Sweden, mid-gut, plain **j** ♂ **k** ♀ **l**, **m** *Gyrinus opacus*, Greenland, plain **l** ♂, mid-gut **m** ♀, ovary **n**, **o** *Gyrinus natator*, ♂, Lough Briskeen, mid-gut **n** plain, **o** the same nucleus C-banded **p–s** *Gyrinus substriatus*, ♂, testis, plain **p**, **q** Kennington **r, s** Isle of Lewis **t, u** *Gyrinus suffriani*, ♂, Catfield Fen, mid-gut, Giemsa stained **t** plain, **u** with spontaneous C-type banding **v**, **w** *Gyrinus urinator*, ♂, Tilford, testis **v** plain **w** the same nucleus C-banded **x**, **y** *Orectochilus villosus*, ♂, Stonesfield, mid-gut **x** plain, **y** the same nucleus C-banded. The scale line to the right of the autosome rows of **u, v** represents 5 μm. The vertical lines on the left-hand side link karyotypes of the same species.

**Figure 6.**
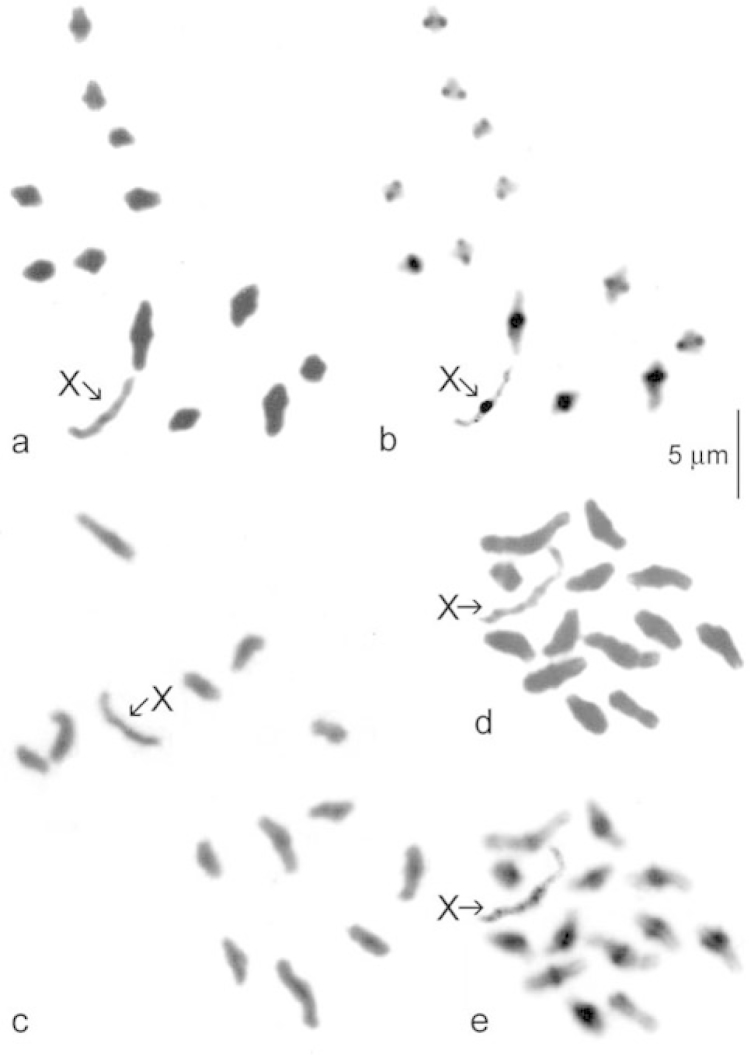
Meiosis, first metaphase from testis. **a, b** *Gyrinus minutus*, **a** plain (Giemsa stained) **b** the same nucleus C-banded **c** *Gyrinus substriatus*, Isle of Lewis, plain (Giemsa stained) **d, e** *Gyrinus urinator* **d** plain (Giemsa stained), **e** the same nucleus C-banded. The scale line represents 5 μm.

Published information: none. Mitotic chromosomes, arranged as a karyotype, are shown in Fig. 5a (plain, Giemsa-stained). Autosome pair 1 has a RCL of about 14.5, and the RCLs decrease fairly evenly along the karyotype to about 5.5 in pairs 7–13. Pair 10 has an obvious secondary constriction. Most of the autosomes are metacentric to submetacentric, with pairs 9 and 12 approaching subacrocentric. The X chromosome, clearly the longest in the nucleus, has a RCL of about 20 and is metacentric. This is the longest X chromosome encountered in the present study. Meiotic chromosomes (first division, diakinesis) are shown in Fig. 6a (plain, Giemsa-stained) and b (C-banded). The single X chromosome is clearly recognisable, as are the centromeric C-bands on all the chromosomes. Although the unpaired X chromosome appears less condensed than the autosomal bivalents, its length is in good agreement with that shown in the karyotype obtained from mitotic chromosomes (Fig. 5a). One autosomal bivalent is missing from this preparation.

#### Subgenus *Gyrinus* s. str.

##### *Gyrinus caspius* Ménétriés, 1832

Published information: 2n = 26 + X0 (♂), karyotype: Saxod and Tetart, 1967. Mitotic chromosomes, arranged as a karyotype, are shown in Fig. 5b (plain, Giemsa-stained) The RCLs of the autosomes range from about 11.5 to about 5.5. Most are metacentric to submetacentric, with pair 3 subacrocentric. Pairs 4 and 9 have a secondary constriction in the short arm. The X chromosome is the longest in the nucleus, RCL about 13.5, and is metacentric. This contradicts Saxod and Tetart who claimed it was the shortest.

##### *Gyrinus paykulli* Ochs, 1927

Published information: n = 13 + X (♂): Saxod and Tetart, 1967; 2n = 26 + X0 (♂), karyotype: Tetart and Saxod 1968. Mitotic chromosomes, arranged as a karyotype, are shown in Fig. 5c (plain, Giemsa-stained), d (partially C-banded, morphology of the chromosomes still clear) and e (the same nucleus as d, fully C-banded but the morphology of some chromosomes lost). All the chromosomes are metacentric with the RCLs of the autosomes decreasing fairly evenly from about 10–6. The X chromosome, RCL about 11.5, is the longest in the nucleus, not the shortest as claimed by Tetart and Saxod. The centromeric C-bands are fairly large, especially on the X chromosome.

##### *Gyrinus distinctus* Aubé, 1836 var. *fairmairei* Régimbart, 1883

Published information: none for var. *fairmairei* but for French *Gyrinus distinctus* 2n = 26 + X0 (♂), karyotype: Saxod and Tetart 1967. Mitotic chromosomes, arranged as karyotypes, are shown in Fig. 5f, g. The RCLs of the autosomes range from about 10.5–5.5, with a fairly even decrease along the karyotype. Pair 10 appears more or less subacrocentric, but the others are all more or less metacentric. The X chromosome, RCL about 14, is clearly the longest in the nucleus.

##### *Gyrinus marinus* Gyllenhal, 1808

Published information: none. 2n = 26 + X0 (♂). Mitotic chromosomes, arranged as karyotypes, are shown in Fig. 5h (plain, Giemsa-stained) and i (C-banded). The RCLs of the autosome range from about 13.5–6, with fairly sharp decreases between pair1 and pairs 2 and 3 (RCLs about 9.5) and between 3 and pair 4 (RCL about 7.7), then a smaller and more gradual decrease to pair 13. All the autosomes are metacentric except for pairs 11 and 12, which are subacrocentric. The X chromosome, RCL about 16, is clearly the longest in the nucleus and is submetacentric. All the chromosomes have small centromeric C-bands.

##### *Gyrinus opacus* C.R. Sahlberg, 1819

Published information: none. 2n = 26 +X0 (♂), XX (♀). Mitotic chromosomes, arranged as karyotypes, are shown in Fig. 5j–m. The RCLs of the autosomes range from about 10–6.5, with a fairly even decrease along the karyotype. All are metacentric, with some variation in centromere position, and pair 2 has a secondary constriction in the long arm. The X chromosome, RCL about 12, is the longest in the nucleus and is metacentric. The mid-gut preparations show the chromatids narrow and well separated except at the centromere while the one from ovary (Fig. 5m) shows the chromatids wider and closer together. There is no detectable difference between Swedish and Greenland material.

##### *Gyrinus natator* (Linnaeus, 1758)

Published information: none. 2n = 26 + X0 (♂). Mitotic chromosomes, arranged as karyotypes, are shown in Fig. 5n (plain, Giemsa-stained) and o (C-banded). The RCLs of the autosomes range from about 10.5–5.5, with a fairly even decrease in length along the karyotype. All the autosomes are metacentric, with pairs 2, 6, 8 and 10 approaching submetacentric. The metacentric X chromosome, RCL about 12.5, is the longest in the nucleus. All the chromosomes have moderate centromeric C-bands.

##### *Gyrinus substriatus* Stephens, 1869

Published information: 2n–26 + X0 (♂): Saxod and Tetart 1967; Tetart and Saxod 1968; karyotype: Tetart and Saxod 1968. Mitotic chromosomes, arranged as karyotypes, are shown in Fig. 5p–s. The RCLs of the autosomes range from about 11–5.5, with a fairly even decrease along the karyotype. Pairs 1, 2, 5, 8 and 13 are evenly metacentric, pairs 3, 4, 6 and 7 are either subacrocentric or on the border with submetacentric, and pairs 9–11 are submetacentric. The X chromosome, RCL about 12, appears to be the longest in the nucleus and is metacentric with a secondary constriction on the long arm, this not always distinct. Diakinesis of first division of meiosis (Fig. 6c) shows the unpaired X chromosome slightly longer than the longest autosomal bivalent. This contradicts Tetard and Saxod who list it as the shortest in the nucleus.

##### *Gyrinus suffriani* Scriba, 1855

Published information: 2n = 26 + X0 (♂): Saxod and Tetart 1967. Mitotic chromosomes, Giemsa-stained and arranged as karyotypes, are shown in Fig. 5t, u. The chromosomes shown in Fig. 5t appear uniformly stained, but those in Fig. 5u show spontaneous C-type banding. These bands appear very large and heavy and may be more extensive than the true C-bands. The RCLs of the autosomes range from about 11–6, with an even decrease from pair 1 to pair 4 (RCL about 7.5), then pairs 5–12 all have the RCL about 7 and pair 13 has it about 6. All except the submetacentric pair 2 are metacentric, and pair 4 has a secondary constriction in its short arm. The metacentric X chromosome, RCL about 13, is the longest in the nucleus, not the shortest as claimed by Saxod and Tetart.

##### *Gyrinus urinator* Illiger, 1807

Published information: none. 2n = 26 + X0 (♂), XX (♀). Mitotic chromosomes, arranged as karyotypes, are shown in Fig. 5v (plain. Giemsa-stained) and w (C-banded). The RCLs of the autosomes range from about 10.5–6, with an even decrease along the karyotype. Pairs 1–5, 9–11, and 13 are metacentric (though one replicate of pair 13 appears very small and may have a deletion), pairs 6–8 are submetacentric and pair 12 appears acrocentric. The metacentric X chromosome, RCL about 12, is the largest in the nucleus. C-banding shows discrete centromeric C-bands on autosome pairs 1–4, while pairs 5 –11, and 13 appear very extensively heterochromatic. The acrocentric autosome pair 12, and the X chromosome, appear to lack C-bands. Diakinesis of first division of meiosis is shown in Fig. 6d (plain, Giemsa-stained) and e (C-banded). This confirms the discrete centromeric C-bands on the larger autosomes and the much heavier ones on the shorter autosomes. Autosome 12 is shown to have a small terminal C-band and the long unpaired X chromosome no C-band at all.

### *Orectochilus* Dejean, 1833

#### *Orectochilus villosus* (Müller, 1776)

Published information: none. 2n = 32 + X0 (♂), XX (♀). Mitotic chromosomes, arranged as karyotypes, are shown in Fig. 5x (plain, Giemsa-stained) and y (C-banded). The karyotype comprises 3 pairs of long autosomes (RCLs about 14–12) metacentric with completely heterochromatic long arms, 1 pair of shorter ones, RCL about 9.5, with a centromeric C-band and a further band on the distal part of the long arm, 2 smaller pairs, RCLs about 7 and 6, with fairly heavy centromeric C-bands, and 10 pairs of shorter autosomes, RCLs 5–3, with small centromeric C-bands. The X chromosome, RCL about 12, is the only long chromosome with a discrete centromeric C-band but otherwise euchromatic. Two C-banded incomplete nuclei obtained from a female both show two such chromosomes, confirming that this is the X chromosome.

## Discussion

### Comparison of the data presented here with those of Saxod and Tetart

Saxod and Tetart (1967, 1968) used only testis as a source of chromosome preparations. Their papers give photographs of chromosomes of five *Gyrinus* species. They presented karyotypes of four of these, and in these cases prepared drawings of the chromosomes *in situ* from the relevant photographs. These drawings were then used to pair up the chromosomes to prepare karyotypes. Before giving a species by species comparison of the data, one general point should be noted: Saxod and Tetart’s illustrations consistently show secondary constrictions on various chromosomes which are more numerous and more distinct than in our material. This appears to be associated with the spreading of partially fixed chromosomes in their technique as against living material in ours.

**Figure 7.**
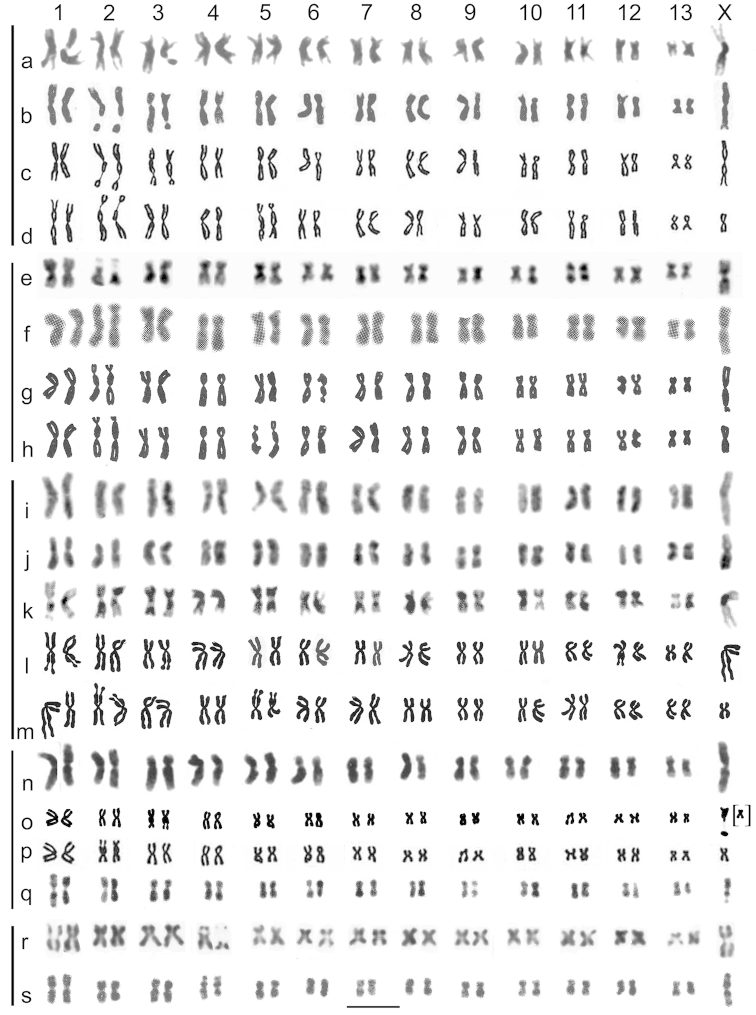
Mitotic chromosomes of *Gyrinus* spp, arranged as karyotypes, to compare the present results with those of Saxod and Tetart (1967) and Tetart and Saxod (1968). **a–d** *Gyrinus caspius*, **a** present material (Fig. 5b) **b** Saxod, Tetart, photograph (Plate 1B) **c** idem, drawing (Fig. 2), arranged as Fig. 5b, d the same drawing as arranged by Saxod, Tetart **e–h** *Gyrinus paykulli*, **e** present material (Fig. 5c) **f** Tetart and Saxod (1968), photograph (Plate1A) **g** idem, drawing, (Fig. 1), arranged as **e, h** the same drawing as arranged by Tetart, Saxod **i, j** *Gyrinus distinctus fairmairei*, present material (Fig. 5f, g) **k–m** *Gyrinus distinctus distinctus* from Saxod and Tetart (1967) **k** photograph (Plate 1A) **l** idem, drawing (Fig. 1), arranged as the present *Gyrinus distinctus fairmairei* (**i, j**) **m** the same drawing as arranged by Saxod, Tetart **n–q** *Gyrinus substriatus* **n** present material (Fig. 5q) **o** drawing by Tetart, Saxod (Fig. 2) but with the X chromosome taken from their photograph (idem, Plate 1C), arranged as present material (**n**) **p** the same drawing as arranged by Tetart, Saxod. The partial X chromosome is placed as the right-hand replicate of chromosome 13 **q** karyotype prepared from Saxod, Tetart (Plate 1D) **r, s** *Gyrinus suffriani* **r** present material (Fig. 5t) **s** karyotype prepared from Saxod and Tetart 1967 (Plate 1C). The horizontal scale-line represents 5 μm. The vertical lines on the left hand side link karyotypes of the same species.

*Gyrinus caspius*. Fig. 7a shows the karyotype presented here while Saxod and Tetart’s photograph and drawing are shown arranged in accordance with our karyotype in Fig. 7b, c and their drawing according to their arrangement is shown in Fig. 7d. The karyotypes shown in Fig. 7b, c show an obvious mismatch in pair 12, also present, though less marked, in Fig. 7a, while the arrangement in Fig. 7d shows mismatches in pairs 1, 4 and 11. The cause of these apparent mismatches is either different degrees of condensation between the replicates of a chromosome pair, if the pairing is correct, or a mispairing if it is not. Different degrees of condensation between the two replicates of a chromosome pair are a frequent occurrence which has to be contended with when assembling karyotypes. In the present case the appearance of the X chromosome in Fig. 7a is strikingly different from the two replicates of autosome pair 1 and is sufficient to show that this attribution is correct, especially as it is consistent with data obtained from other species where the unpaired X chromosome is shown in diakinesis of first division of meiosis.

*Gyrinus paykulli*. Fig. 7e shows the karyotype presented here, while Tetart and Saxod’s material is shown in Fig. 7f (photograph) and g (drawing), arranged according to our interpretation, and the drawing according to their interpretation is shown as Fig. 7h. In this case our interpretation of Tetart and Saxod’s pictures shows minor mismatches in pairs 1 and 9, while their arrangement shows more serious mismatches in pairs 2 and 5, a different minor mismatch in pair 1 and the same minor mismatch in pair 9. Saxod and Tetart (1967) figure diakinesis of first division of meiosis, and this photograph is shown in Fig. 8c. The unpaired X chromosome is clearly visible and about as long as the longest bivalents. This agrees with our interpretation.

*Gyrinus distinctus*. Fig. 7i, j shows the karyotype presented here for var. *fairmairei*, while Saxod and Tetart’s material is shown to the same arrangement if Fig. 7k (photograph) and l (drawing), with the drawing as arranged by Saxod and Tetart shown in Fig. 7m. Our arrangement shows no serious mismatches between replicates of a chromosome pair, but minor mismatches in pairs 12 and 13, while Saxod and Tetart’s arrangement shows serious mismatches in pairs 1 and 2, but no mismatches in pairs 12 and 13. The other thing to note is that the detailed sequence of chromosome dimensions obtained from our material agrees very closely with those obtained when Saxod and Tetart’s data are arranged in the same way. These data give no support to placing var. *fairmairei* as a species separate from *Gyrinus distinctus*.

*Gyrinus substriatus*. Fig. 7n shows the karyotype presented here, while Fig. 7o shows Tetart and Saxod’s drawing arranged in the same way, and Fig. 7p shows the drawing as arranged by Tetart and Saxod. Finally, Fig. 7q shows a karyotype assembled according to our arrangement, from the photograph given by Saxod and Tetart. This species apparently gave Saxod and Tetart some difficulty. They chose not to assemble a karyotype from their 1967 photograph, perhaps because they thought that one of the chromosomes, lying at some distance from the others, might not belong to the same nucleus. The 1968 photograph they used is shown as their Plate 1 C and the drawing prepared from it as Fig. 2. The chromosomes as shown in the photograph are too condensed and clumped to allow assembly of a karyotype, but the drawing seems adequate. However, the photograph shows the X chromosome (as here interpreted) with a very large secondary constriction but in the drawing the distal part of the chromosome, beyond the constriction, is omitted. The chromosomes are all very condensed and neither arrangement of their material shows any obvious mismatches. However, *Gyrinus substriatus* is a species for which we have a preparation showing the unpaired X chromosome at diakinesis in the first division of meiosis. This leaves no doubt that this is a large chromosome. Tetart and Saxod (1968, Plate 1 E) show a first meiotic metaphase for this species. The chromosomes are very condensed and not clear, but what appears to be the X chromosome is as long as the longest autosomal bivalents. The karyotype shown in Fig. 7q shows the same secondary constriction in the X chromosome as that shown in Fig. 7o, p, but shows the X chromosome relatively shorter than in our material (Fig. 7n), though this is less apparent when the more condensed (shorter chromosomes) Isle of Lewis material (Fig. 5r, s) is considered.. There is also an obvious mismatch in pair 1. It seems likely that the apparent difference in X chromosome length between our material and Saxod and Tetart’s results from the more condensed chromosomes in their material, with the mismatch in pair 1 in Fig. 7q resulting from uneven condensation.

*Gyrinus suffriani*. The karyotype presented from our material is shown in Fig. 7r and one assembled from the photograph given by Saxod and Tetart (1967) is shown in Fig. 7s. The two agree very closely. Saxod and Tetart did not give a karyotype for *Gyrinus suffriani*, only the chromosome number. The X chromosome is the longest in both Figures and shows a secondary constriction in the long arm of Saxod and Tetart’s material, comparable with that in *Gyrinus substriatus*. The secondary constriction in the short arm of chromosome 2 is apparent in both our material and that of Saxod and Tetart.

### Interspecies differences

The data presented here show that the 10 species of *Gyrinus* discussed here all have broadly similar karyotypes, with 13 pairs of autosomes X0 sex chromosomes, and the X chromosome the longest in the nucleus. There are often small differences between the chromosomes of different species. Thus the relative length of the X chromosome of *Gyrinus minutus* is about 1.5 times that of the longest autosome, a bigger difference than in any of the other species studied.

There are two species-groups among the studied species. *Gyrinus caspius* and *paykulli* are conspicuously elongate parallel-sided beetles, though with very different aedeagi. Unfortunately the chromosomal material presented here is inadequate to show interspecies differences. The second group, *Gyrinus natator*, *substriatus* and *suffriani*, does show some interspecies differences. In *Gyrinus natator* the more or less submetacentric chromosome 2 is clearly longer than metacentric chromosome 3, while in *Gyrinus substriatus* metacentric chromosome 2 is longer than submetacentric 3, though only slightly so. The X chromosome is only slightly longer than chromosome 1 in these species. In *Gyrinus suffriani* the X chromosome is more clearly longer than chromosome 1, chromosome 4 has a distinct secondary constriction in its short arm, and there is more marked decrease in length between pairs 4 and 5.

There are two cases where conspecific material from widely separated localities shows no chromosomal difference. This is true of Swedish and Greenland *Gyrinus opacus*, with the Greenland material belonging to the form which lacks elytral reticulation, and of Kuwaiti and French *Gyrinus distinctus*, with the Kuwaiti material belonging to var. *fairmairei* with a yellow underside as against the largely black underside of the French material.

*Gyrinus urinator* is the only species with acrocentric chromosomes–chromosome 12.

The chromosomes of *Orectochilus villosus* agree with those of *Gyrinus* spp. in having an X0 sex chromosome system and a relatively long X chromosome. However, they differ in having 16 pairs of autosomes as against 13 in *Gyrinus*, and in having the three longest pairs with heterochromatic long arms, then a fairly long pair with a largely heterochromatic longer arm, and the remaining pairs short to very short with small centromeric C-bands, ranging from metacentric to subacrocentric.
